# HIPEC as Up-Front Treatment in Locally Advanced Ovarian Cancer

**DOI:** 10.3390/cancers16203500

**Published:** 2024-10-16

**Authors:** Michail Karanikas, Konstantinia Kofina, Dimitrios Kyziridis, Grigorios Trypsianis, Apostolos Kalakonas, Antonios-Apostolos Tentes

**Affiliations:** 1 1st Department of Surgery, School of Medicine, Democritus University of Thrace, 68100 Alexandroupolis, Greece; michaelkaranikas@yahoo.gr; 2 Department of Surgical Oncology, Peritoneal Surface Malignancy Program, EUROMEDICA Kyanous Stavros, 54644 Thessaloniki, Greece; dkyziridis@gmail.com (D.K.); tolistentes@gmail.com (A.-A.T.); 3 Department of Medical Statistics, School of Medicine, Democritus University of Thrace, 68100 Alexandroupolis, Greece; gtryps@med.duth.gr; 4 Department Anesthesiology, EUROMEDICA Kyanous Stavros, 54644 Thessaloniki, Greece; apostoliskal@gmail.com

**Keywords:** ovarian cancer, cytoreductive surgery, HIPEC, survival

## Abstract

Purpose: The main objective of the study is to evaluate the effect of hyperthermic intraperitoneal chemotherapy (HIPEC) in the treatment of naïve ovarian cancer women undergoing complete or near-complete cytoreduction by assessing the overall survival, the disease-specific survival, and the disease-free survival. The secondary objective is the identification of prognostic indicators of survival and recurrence of these patients. Patients—Methods: Retrospective study of treatment in naïve women with locally advanced ovarian cancer treated with cytoreductive surgery (CRS) and HIPEC and compared with those who were treated with cytoreduction alone. Clinicopathologic variables were correlated to overall survival, disease-specific survival, and disease-free survival using Kaplan–Meier method, and the multivariate Cox proportional hazards regression models. Results: 5- and 10-year overall survival, disease-specific survival, and disease-free survival rates were significantly higher in patients treated with CRS and HIPEC. These patients were 67% less likely to die from any cause (adjusted hazard ratio, aHR = 0.33, *p* = 0.001), 75% less likely to die from cancer (aHR = 0.25, *p* = 0.003), and 46% less likely to develop recurrence (aHR = 0.54, *p* = 0.041) compared to patients treated with CRS alone. Moreover, the poor performance status (aHR = 2.96, *p* < 0.001), the serous carcinomas (aHR = 0.14, *p* = 0.007), and the morbidity (aHR = 6.87, *p* < 0.001) were identified as independent indicators of poor overall survival. The degree of differentiation (aHR = 8.64, *p* = 0.003) was identified as the independent indicator of disease-specific survival (aHR = 4.13, *p* = 0.002), while the extent of peritoneal carcinomatosis (aHR = 2.32, *p* < 0.001) as the independent indicator of disease-free survival. Conclusions: Treatment in naïve patients with locally advanced ovarian cancer undergoing CRS plus HIPEC appears to have improved overall, disease-specific, and disease-free survival.

## Simple Summary

This study evaluated the effect of hyperthermic intraperitoneal chemotherapy with complete or near-complete cytoreduction on naïve ovarian cancer women; 5- and 10-year overall survival, disease-specific survival, and disease-free survival rates were significantly higher in these patients. These patients were also 67% less likely to die from any cause, 75% less likely to die from cancer, and 46% less likely to develop recurrence compared to patients that were treated with cytoreductive surgery alone. These favorable results highlight the need for future randomized controlled trials in order to further determine the effect of hyperthermic intraperitoneal chemotherapy.

## 1. Introduction

Ovarian cancer is the sixth most common malignant tumor in female patients with an unfavorable prognosis in Western Europe. The lack of specific symptoms even in the locally advanced stage makes early diagnosis almost impossible. The diagnosis is established either incidentally or in late stages when the disease is associated with peritoneal carcinomatosis [1]. Cytoreductive surgery (CRS) in combination with systemic chemotherapy is still considered the standard treatment. Ovarian cancer is one of the most chemosensitive neoplasms and approximately 80% of women respond to CRS and adjuvant chemotherapy but 80% of them relapse and need further treatment [2]. Complete cytoreduction without a macroscopically visible residual tumor has been documented as the most significant prognostic indicator of survival [3]. The implementation of standard peritonectomy procedures [4] has been very helpful in achieving complete or near-complete cytoreduction [5]. Prospective randomized trials have shown that neo-adjuvant chemotherapy (NACT) does not improve overall survival [6,7] but is helpful in achieving complete tumor resection (R_0_) more frequently [8] because the extent of the tumor load may be significantly reduced. The use of NACT has been definitely established only for stage IV ovarian cancer patients for whom it should be the standard of care [9].

Hyperthermic intraperitoneal chemotherapy (HIPEC) has been used since 1994 in ovarian cancer. The indication for the use of HIPEC after CRS in ovarian cancer was initially implemented for recurrent cancer [10]. Nevertheless, a few years later, it was used even as an up-front treatment [11,12,13]. Although two prospective randomized trials have shown that HIPEC offers significant survival benefit in women with ovarian cancer [14,15], the method has not been established as a standard treatment modality. There are many ongoing trials attempting to identify the role of HIPEC in ovarian cancer. In addition, it has not been definitely established if NACT should always be used in newly diagnosed patients or CRS plus HIPEC has a distinct role in the treatment. A consensus of PSOGI members (Peritoneal Surface Oncology Group International) was conducted to the conclusion that if the surgeon may perform a CC-0 or CC-1 operation, CRS plus HIPEC should be preferred instead of NATC.

HIPEC is an efficient method for the eradication of the microscopic tumor. It may penetrate up to 2–3 mm. Therefore, it may be given in patients that have undergone CC-0 or CC-1 surgery. It has no effect in patients that have undergone CC-2 or CC-3 surgery. Other contraindications for HIPEC administration are white blood cell count < 4000, thrombocytes < 100.000 blood urea level > 50 mg/dL, creatinine level > 1.5 mg/dL, and abnormal liver function tests.

The primary objective of the study is the evaluation of the effect of HIPEC in the treatment of naïve ovarian cancer patients who underwent complete or near-complete cytoreduction by assessing the overall survival, the disease-specific, and the disease-free survival. The secondary objective is the identification of the prognostic indicators of survival and recurrence of these patients.

## 2. Patients and Methods

The files of the patients with newly diagnosed ovarian cancer from 2000 until 2020 were retrospectively reviewed. The files of patients that had previously undergone therapeutic surgery or of those who had undergone incomplete cytoreduction were excluded from the study.

The diagnosis was possible with physical examination, thoracic and abdominal CT-scan or MRI or very infrequently with PET-CT scan, hematologic and biochemical examinations, tumor markers (CEA, CA-125), and in some cases with biopsy. The performance status was assessed according to the Karnofsky performance scale. The ASA classification was made by the responsible anesthesiologist.

All patients underwent cytoreductive surgery with the intent of performing complete or near-complete tumor resection. A mid-line incision extending from the xiphoid process to the symphysis pubis and a Thompson self-retaining retractor gave full access to the abdomen. The extent of previous surgery was assessed using the prior surgery score (PSS-score). Patients with no previous surgery were indicated as PSS-0, while those that underwent biopsy were indicated as PSS-1. The extent and distribution of the tumor load was assessed according to the peritoneal cancer index (PCI). The tumor volume was assessed as small and large volume. Implantations with maximal diameter < 0.5 cm were considered as small-volume tumors, while implantations with maximal diameter > 0.5 cm or confluent of any diameter were considered as large-volume tumors. After surgical resection of the tumor, the completeness of cytoreduction was assessed using the CC-score. CC-0 surgery indicated patients without macroscopically visible residual tumors. CC-1 surgery indicated patients with residual tumors that had maximal diameter < 0.25 cm. CC-2 indicated residual tumors > 0.25 cm and <2.5 cm, while CC-3 indicated residual tumors > 2.5 cm. CC-2 and CC-3 cytoreductive operations were considered incomplete cytoreductions [16,17].

After tumor resection, HIPEC was performed for 90 min at 42–43 °C using the open abdominal (Coliseum) technique. A heater circulator with two roller pumps, one heat exchanger, one reservoir, an extracorporeal system of two inflow and two outflow tubes, and four thermal probes was used for HIPEC (Sun Chip, Gamida Tech, France). A prime solution of 2–3 L of Normal Saline or Ringer’s Lactate was instilled prior to the administration of the cytostatic drug and as soon as the mean abdominal temperature reached 40 °C, the cytostatic drugs were instilled in the abdomen. Cis-platinum (50 mg/m^2^) and doxorubicin (15 mg/m^2^) were used in HIPEC and ifosphamide (1300 mg/m^2^) plus Mesna (260 mg/m^2^) were given intra-venously. The dose of Mesna was used two more times after 4 and 8 h to prevent the side effects of ifosphamide in the bladder. The reconstruction of the continuity of the gastrointestinal tract was made after the completion of HIPEC. Proximal stoma defunctioning was always performed in those cases in which more than two anastomoses should be protected. All patients remained in the ICU for at least 24 h until hemodynamic stabilization. The complications were carefully recorded. All patients received adjuvant chemotherapy with platinum and taxanes derivatives one month after initial treatment.

All patients were followed up every 3–4 months for the first year after initial treatment, and every 6 months later. The follow-up included physical examination, thoracic and abdominal CT-scan or MRI or PET-CT scan, hematologic and biochemical examinations, and tumor markers (CEA, CA-125). Recurrences and the sites of recurrence were recorded in detail.

All specimens were examined in detail and the sub-type of the tumor, the degree of differentiation, the number of the resected and the infiltrated lymph nodes were recorded, as well as the site and the depth of implantation at any other organ.

The age, the performance status, that was assessed according to the Karnofsky performance scale [16], the ASA (American Society of Anesthesiologists) classification stage, the extent and distribution of the peritoneal spread, the extent of previous surgery, the completeness of cytoreduction, the tumor volume, the use of HIPEC, histopathologic details, the recurrences and the sites of recurrence, the morbidity and in-hospital mortality, the need for blood and/or fresh frozen plasma (FFP) transfusion, and the days of hospitalization were recorded and correlated to survival.

### Statistical Analysis

Statistical analysis of the data was performed using IBM Statistical Package for Social Sciences (SPSS), version 19.0 (IBM Corp., Armonk, NY, USA). The chi-square test was used to evaluate any potential association between HIPEC and the clinicopathological parameters. As indicators of survival, the overall survival (OS; time from cancer diagnosis to death from any cause), the disease-specific survival (DSS; time from cancer diagnosis to death from cancer), and the disease-free survival (DFS; time from the primary treatment of cancer that the patient survives without any signs or symptoms of that cancer) were investigated. Survival rates were calculated with the Kaplan–Meier method, and the statistical difference between survival curves was determined with the log-rank test. Multivariate Cox proportional hazards regression analysis, using a backward selection approach, was performed to explore the independent effect of HIPEC and other clinicopathological parameters on survival indicators. All tests were two-tailed, and statistical significance was considered for *p* values < 0.05.

## 3. Results

From 2000 until 2020, 151 female patients, mean age 61.95 ± 12.34 years, with peritoneal carcinomatosis of epithelial ovarian cancer that did not receive NACT underwent complete (CC-0) or near-complete (CC-1) cytoreduction. The clinicopathologic characteristics of the patients are listed in Table 1. Seventy-nine patients were treated with CRS plus HIPEC, while 72 were treated with CRS alone. Patients treated with CRS plus HIPEC were younger in age (*p* = 0.033), were in better performance status (*p* = 0.021), presented increased morbidity (*p* = 0.001), and underwent more frequently retroperitoneal lymph node resection (*p* < 0.001).

### 3.1. Overall Survival

**Figure 1:**
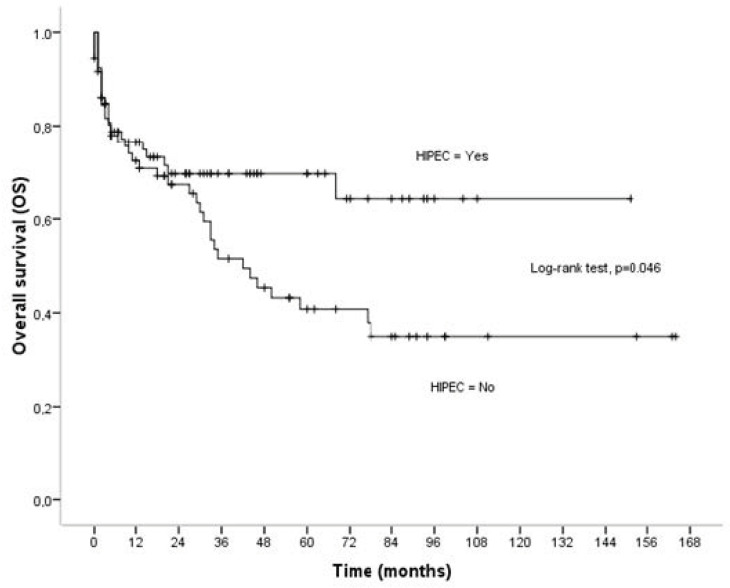
Overall survival (OS) in relation to HIPEC.

Follow-up data were available for all patients. The median follow-up time was 22 months (0–164). Sixty patients (39.7%) died from any cause during follow-up. The mean and median survival times were 89.44 ± 7.18 months (95% CI = 75.36–103.52), and 77 months, respectively (Figure 1). The advanced age (*p* < 0.001), poor performance status (*p* < 0.001), poor ASA class (*p* < 0.001), the large-volume tumor (*p* = 0.013), high PCI (*p* < 0.001), the serous carcinomas (*p* < 0.001), the low degree of differentiation (*p* < 0.001), and the morbidity (*p* < 0.001) were found to be related to poor overall survival (Table 2). The 5- and 10-year survival rate for patients treated with CRS plus HIPEC was 69.64 ± 5.51%, and 64.28 ± 7.23%, respectively. The 5- and 10-year survival rate for patients treated with CRS alone was 40.79 ± 6.76%, and 34.96 ± 6.94%, respectively. The overall survival of patients who were not treated with HIPEC was worse compared to the survival of patients treated with HIPEC (*p* = 0.046). The mean survival time was 102.51 ± 8.71 months (95% CI = 85.43–119.59 months) for patients treated with HIPEC, and 73.90 ± 9.37 months (95% CI = 55.54–92.26 months) for patients treated with CRS alone (Table 2 Figure 1). The number of patients who died was significantly smaller in patients treated with HIPEC compared to patients treated with CRS alone (29.1% vs. 51.4%, *p* = 0.005). Multivariate analysis revealed that the use of HIPEC remained a significant independent indicator of improved overall survival; in particular, patients treated with HIPEC were 67% less likely to die from any cause than those who did not receive HIPEC (adjusted hazard ratio = 0.33, 95% CI = 0.17–0.63, *p* = 0.001) (Table 3). The low performance status (aHR = 2.96, 95% CI = 1.70–5.17, *p* < 0.001), the non-serous carcinomas (aHR = 0.14, 95% CI = 0.03-0.59, *p* = 0.007), and morbidity (aHR = 6.87, 95% CI = 3.49–13.52, *p* < 0.001) were also found to be independent predictors of overall survival (Table 3).

### 3.2. Disease-Specific Survival

**Figure 2:**
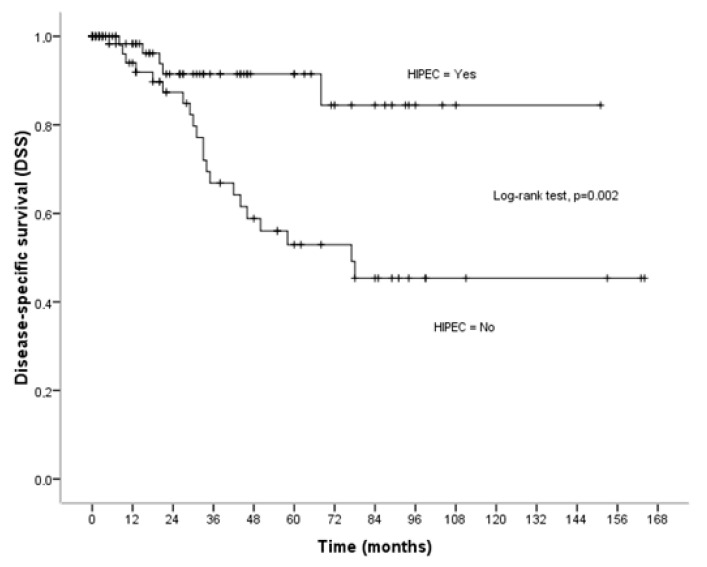
Disease-specific survival (DSS) in relation to HIPEC.

During the follow-up period, twenty-six patients (17.2%) died from cancer. Among the entire cohort, the mean disease-specific survival time was 115.83 ± 7.80 months (95% CI = 100.53–131.12 months) although the median survival time was not reached. The older age (*p* = 0.043), the low performance status (*p* < 0.001), the high ASA class (*p* = 0.001), the serous carcinomas (*p* = 0.005), and carcinomas with a low degree of differentiation (*p* < 0.001) were found to be related to poor disease-specific survival (Table 2). The 5- and 10-year disease-specific survival rates for patients treated with CRS plus HIPEC were 91.48 ± 4.12% and 84.44 ± 7.76%. For patients treated with CRS alone, the 5- and 10-year survival rates were 52.92 ± 8.06% and 45.36 ± 8.50%, respectively (*p* = 0.002). Patients who were not treated with HIPEC had worse disease-specific survival (*p* = 0.002). The mean survival time was 133.66 ± 7.70 months (95% CI = 118.57–148.75 months) for patients treated with CRS+HIPEC, and 95.16 ± 10.53 months (95% CI = 74.51–115.80 months) for patients treated with CRS alone (Figure 2). The number of patients that died because of cancer was significantly smaller in the HIPEC group compared to the non-HIPEC group (6.3% vs. 29.2%, *p* < 0.001). Multivariate analysis revealed that the use of HIPEC (aHR = 0.25, 95% CI = 0.10–0.63, *p* = 0.003), and the high differentiated carcinomas (aHR = 8.64, 95% CI = 2.03–36.73, *p* = 0.003) were identified as independent indicators for poor disease-specific survival (Table 4). In particular, patients treated with HIPEC were 75% less likely to die from cancer than those treated with CRS (Table 4).

### 3.3. Disease-Free Survival

**Figure 3:**
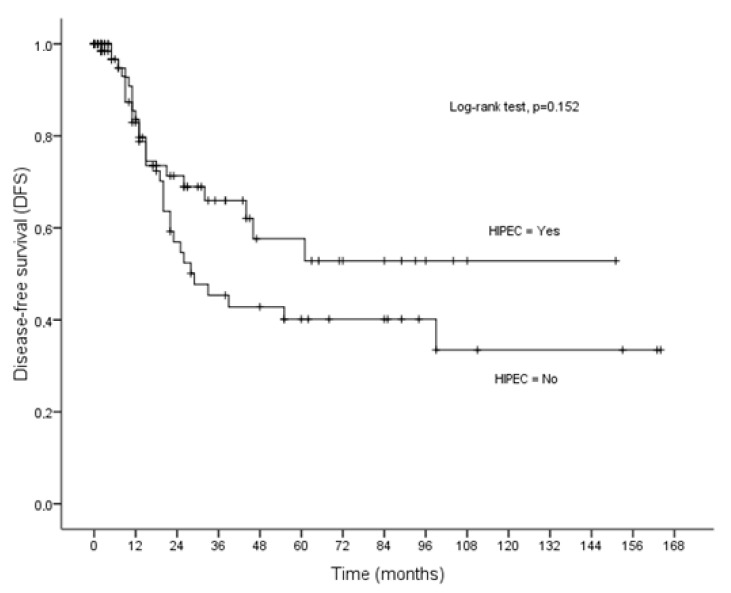
Disease-free survival (DFS) in relation to HIPEC.

Disease recurrence was recorded in 49 patients (32.5%). The mean and median disease-free survival time was 84.78 ± 8.10 months (95% CI = 68.90–100.66) and 77 months, respectively. The performance status (*p* = 0.003), the ASA stage (*p* = 0.034), the tumor volume (*p* = 0.049), the histopathologic sub-type (*p* < 0.001), the degree of differentiation (*p* < 0.001), and the PCI (*p* = 0.002) were found to be related to poor disease-free survival (Table 2). For patients treated with CRS plus HIPEC, the 5-, and 10-year disease-free survival rates were 57.63 ± 8.18% and 52.82 ± 8.79%, respectively, while for patients treated with CRS alone, the 5- and 10-year disease-free survival was 40.14 ± 7.38% and 33.45 ± 8.67%, respectively (*p* = 0.152). From univariate analysis, no significant difference was observed between CRS+HIPEC and CRS in disease-free survival (*p* = 0.152). The mean disease-free survival time was 91.29 ± 10.50 months (95% CI = 70.71–111.88) for patients treated with CRS plus HIPEC, and 73.49 ± 10.56 months (95% CI = 52.79–94.20) for patients treated with CRS alone (Figure 3). However, the recurrence rate for patients treated with HIPEC was significantly lower compared to those treated with CRS alone (25.3% vs. 40.3%, *p* = 0.050). Multivariate analysis revealed that the use of HIPEC (aHR = 0.54, 95% CI = 0.30–0.97, *p* = 0.041), the low degree of differentiation (aHR = 4.13, 95% CI = 1.69-10.13, *p* = 0.002), and the extent of peritoneal carcinomatosis (PCI) (aHR = 2.32, 95% CI = 1.1–4.89, *p* < 0.001) were independent indicators of disease-free survival (Table 5). In particular, patients treated with HIPEC were 46% less likely to develop recurrence than those treated with CRS (Table 4).

## 4. Discussion

The survival rate for stage III and IV ovarian cancer patients has not improved in the last two decades and so far, the 10-year survival rate has not exceeded 10-15%. Survival may improve if a diagnosis can be achieved in earlier stages or if the relapse may be delayed or prevented. The lack of specific symptoms or specific hematological-biochemical or even radiological examinations makes it impossible for early diagnosis of ovarian cancer. As a consequence, it appears that treatment should be focused in decreasing the incidence of recurrence. The most frequent sites of recurrence in ovarian cancer are the lines of resection, particularly the vaginal stump, and the peritoneal surfaces. Small-volume implantations located in the cul-de-sac of Douglas, on the rectosigmoid, in the left abdominal gutter, or in the remnant of the greater omentum that are not easily visible by first sight or by an inexperienced surgeon are apparently the principal reasons for recurrent disease [17]. The term “recurrent disease” does not accurately describe this pathophysiologic condition, and it should rather be considered as the disease progression of a persistent tumor [18]. The surgical resection of the primary tumor with peritoneal seedings remains the most powerful tool for the treatment of peritoneal malignancies of any origin. Therefore, the completeness of cytoreduction is the most significant prognostic indicator of survival [3]. In our study, although the CC-0 patients treated with HIPEC were found to have better survival compared to CC-1 patients, the difference was not statistically significant because the number of CC-1 operations was very small. It has been shown that surgery alone is not sufficient to provide long-term survival in women with locally advanced ovarian cancer because even if a complete cytoreduction is achieved, microscopic cancer emboli always remain in the abdominal cavity. Treatment with systemic chemotherapy cannot eradicate the microscopic residual tumor which lacks adequate blood supply in contrast to heated intraperitoneal chemotherapy [19]. The peritoneal-plasma barrier makes it difficult for the absorption of high-molecular chemical compounds which remain for long at the peritoneal surfaces before being absorbed in the systemic circulation. Most of the cytotoxic drugs are high-molecular substances and acting synergistically with heat may destroy the microscopic implantantions.

Despite encouraging results concerning the use of HIPEC in ovarian cancer, the European Society of Medical Oncology and the European Society of Gynecologic Oncology have not adopted the method for women with newly diagnosed primary ovarian cancer because adequate evidence is still missing [12]. A recent single-center prospective randomized trial has confirmed the results of previous trials [14,15] identifying the significance of HIPEC in the treatment of patients with ovarian cancer that received NACT [15]. A meta-analysis of randomized trials showed that in primary ovarian cancer interval CRS plus HIPEC and NACT improved significantly the overall survival and the disease-free survival [20]. The results from publications of the international literature are in agreement with the results of the present study. Van Driel et al. showed in a prospective randomized trial that the addition of HIPEC to interval cytoreduction improved the overall survival and the disease-free survival [13]. A few years earlier. Gonzalez-Bayon L et al. showed that CRS plus HIPEC was a promising method for the up-front treatment of locally advanced ovarian cancer [21]. A relatively recent trial reconfirmed these findings [22].

Our findings demonstrate that the proportion of patients who died and were treated with HIPEC was smaller than that of patients who died and had undergone CRS alone. In particular, multivariate Cox regression analysis revealed that patients treated with HIPEC were 67% less likely to die from any cause (adjusted hazard ratio, aHR = 0.33, *p* = 0.001), 75% less likely to die from cancer (aHR = 0.25, *p* = 0.003), and 46% less likely to develop recurrent disease (aHR = 0.54, *p* = 0.041) than those who did not receive HIPEC. Cascales Campos PA et al. showed that HIPEC was a prognostic indicator of recurrence in a recent phase III randomized trial [15]. These findings have been reconfirmed by our study. It appears that the decrease of recurrences contributed significantly in the improvement of overall survival, disease-specific survival, and disease-free survival with the use of HIPEC [15,22,23,24,25]. Two meta-analyses showed that the addition of HIPEC in CRS improved significantly the overall and the disease-free survival [26,27].

The increased intraoperative time and the increased rate of hospital morbidity concerning hematological toxicity or surgical complications have been considered the most profound disadvantages for the integration of HIPEC in an otherwise long surgical operation. Two single-center studies have shown no difference between HIPEC and non-HIPEC groups of patients in regard to morbidity and quality of life [9,10]. Praiss et al. showed that even after secondary cytoreduction, morbidity remained the same by the addition of HIPEC [28]. In our study, the morbidity rate was found to be significantly higher in the HIPEC group in contrast to other studies [13].

The groups in our study were not comparable for age, performance status, retroperitoneal lymph node resection, and recurrences. The HIPEC group included more younger patients than the non-HIPEC group, more patients with better performance status, more patients that underwent retroperitoneal lymph node resection, and the number of the recorded recurrences was smaller. Nevertheless, they were comparable for the ASA stage, the tumor volume, the completeness of cytoreduction, and the extent of the peritoneal disease. There are various ways to control the effect of confounding variables, including randomization, restriction, matching, or multivariate statistical models. Multivariate statistical analysis was used in our study to eliminate the effect of these potential confounders [29].

In our study, it was shown that advanced age, poor performance status, poor ASA stage, large tumors, serous carcinomas, high degree of differentiation, high PCI, and morbidity were adversely related to survival (*p* < 0.05) and only poor performance status, serous carcinomas, and morbidity together with HIPEC, were identified as independent prognostic factors of overall survival. Moreover, advanced age, poor performance status, poor ASA stage, serous carcinomas, high degree of differentiation, and high PCI were significantly related to disease-specific survival (*p* < 0.05), but only the high degree of differentiation remained independent prognostic factors. Finally, poor performance status, low ASA stage, serous carcinomas, high degree of differentiation, and high PCI were found to be related to disease-free survival (*p* < 0.05), but the high degree of differentiation and high PCI remained independent indicators of disease-free survival. The current concept of treatment for the newly diagnosed cases of ovarian cancer consists of cytoreductive surgery alone followed by systemic chemotherapy with or without bevacizumab. This treatment is followed by maintenance therapy with PARP-inhibitors and olaparib-bevacizumab and has resulted in a consensus of clinical oncologists [30].

The strong points of the study are (1) it has been performed by the same surgical and anesthesiological team, (2) the number of the included patients was large, and (3) the surgical methods and the HIPEC method were the same. As a retrospective study, it obtains many biases although all the data were prospectively maintained. However, with the exception of the retroperitoneal lymph node resection, the surgical characteristics were comparable between the two groups. Despite two more different characteristics (the age and the performance status), the results of the study provide much evidence that the integration of HIPEC in CRS may possibly be beneficial for women with ovarian cancer.

## 5. Conclusions

CRS plus HIPEC has shown favorable results considering overall survival, disease-specific, and disease-free survival in our study. Future randomized controlled trials will further determine the effect of HIPEC on survival outcomes, while individualized management of patients with epithelial ovarian cancer with the right combination of treatments is expected to be feasible and most effective.

## Figures and Tables

**Table 1:** Comparison of patient groups that received CRS plus HIPEC versus CRS alone, without preoperative chemotherapy.

	Total	CRS + HIPEC	CRS	*p* Value
Number of patients	151	79	72	
Age				0.033
<65	89 (58.9)	53 (67.1)	36 (50.0)	
≥65	62 (41.1)	26 (32.9)	36 (50.0)	
Performance status				0.021
90–100%	108 (71.5)	64 (81.0)	44 (61.1)	
70–80%	39 (25.8)	13 (16.5)	26 (36.1)	
50–60%	4 (2.6)	2 (2.5)	2 (2.8)	
ASA class				0.067
I	104 (68.9)	61 (77.2)	43 (59.7)	
II	44 (29.1)	17 (21.5)	27 (37.5)	
III	3 (2.0)	1 (1.3)	2 (2.8)	
Tumor volume				0.489
Small	30 (19.9)	14 (17.7)	16 (22.2)	
Large	121 (80.1)	65 (82.3)	56 (77.8)	
CC-score				0.435
CC-0	140 (92.7)	72 (91.1)	68 (94.4)	
CC-1	11 (7.3)	7 (8.9)	4 (5.6)	
Histopathology				0.635
Serous	119 (78.8)	61 (77.2)	58 (80.6)	
Endometrioid	22 (14.6)	11 (13.9)	11 (15.3)	
Mucinous	4 (2.6)	2 (2.5)	2 (2.8)	
Border-line	5 (3.3)	4 (5.1)	1 (1.4)	
Yolk-sac	1 (0.7)	1 (1.3)	0 (0.0)	
Degree of differentiation				0.087
G1	29 (19.2)	16 (20.3)	13 (18.1)	
G2	13 (8.6)	3 (3.8)	10 (13.9)	
G3	109 (72.2)	60 (75.9)	49 (68.0)	
RLND	31 (20.5)	29 (36.7)	2 (2.8)	<0.001
Relapse	49 (32.5)	20 (25.3)	29 (40.3)	0.050
PCI				0.078
0–13	82 (63.9)	36 (45.6)	46 (63.9)	
14–20	40 (26.5)	25 (31.6)	15 (20.8)	
21–39	29 (19.2)	18 (22.8)	11 (15.3)	
Morbidity	47 (31.1)	34 (43.0)	13 (18.1)	0.001

Explanations: RLND = abdonopelvic lymph node resection, PCI = peritoneal cancer index.

**Table 2:** Mean overall survival (OS), disease-specific survival (DSS), and disease-free survival (DFS).

	Overall Survival (OS)		Disease-Specific Survival (DSS)		Disease-Free Survival (DFS)	
	Mean ± SE	*p* Value	Mean ± SE	*p* Value	Mean ± SE	*p* Value
Age		<0.001		0.043		0.215
<65	109.73 ± 8.80		126.71 ± 8.73		93.67 ± 9.37	
≥65	41.16 ± 5.92		64.61 ± 7.08		50.04 ± 7.91	
Performance status		<0.001		<0.001		0.003
90–100%	111.35 ± 7.89		130.65 ± 7.68		93.99 ± 8.85	
<90%	24.30 ± 4.72		42.87 ± 6.42		26.57 ± 4.81	
ASA class		<0.001		0.001		0.034
I	108.30 ± 8.10		129.22 ± 7.96		92.96 ± 9.00	
II–III	31.89 ± 5.66		51.16 ± 7.16		34.96 ± 6.76	
Tumor volume		0.013		0.226		0.049
Small	76.78 ± 6.90		82.19 ± 6.24		71.68 ± 7.73	
Large	81.21 ± 7.79		110.99 ± 8.78		77.42 ± 8.70	
CCC-score		0.092		0.181		0.928
CC-0	92.81 ± 7.47		118.55 ± 7.97		85.29 ± 7.87	
CC-1	38.09 ± 11.94		59.40 ± 13.49		52.67 ± 16.47	
Histopathology		<0.001		0.005		<0.001
Serous	74.07 ± 7.84		99.67 ± 9.22		67.23 ± 8.72	
Other	104.13 ± 4.71		107.48 ± 3.46		98.53 ± 6.72	
Degree of differentiation		<0.001		<0.001		<0.001
G1/G2	146.82 ± 7.70		158.17 ± 4.75		132.83 ± 11.01	
G3	68.42 ± 8.11		95.74 ± 9.88		61.87 ± 9.21	
Lymph node dissection		0.349		0.157		0.428
Conventional	85.19 ± 7.87		110.25 ± 8.65		81.75 ± 9.01	
RLNR	104.90 ± 12.83		136.72 ± 9.57		88.24 ± 16.84	
PCI		<0.001		0.071		0.002
0–13	113.58 ± 8.99		126.14 ± 8.85		97.56 ± 10.17	
14–20	72.43 ± 13.79		103.92 ± 16.72		80.66 ± 16.66	
21–39	26.06 ± 7.45		49.97 ± 11.79		25.28 ± 6.91	
Morbidity		<0.001		0.694		0.213
No	104.32 ± 8.34		114.47 ± 8.48		87.80 ± 9.04	
Yes	55.92 ± 11.44		120.43 ± 16.01		61.98 ± 17.14	
HIPEC		0.046		0.002		0.152
No	73.90 ± 9.37		95.16 ± 10.53		73.49 ± 10.56	
Yes	102.51 ± 8.71		133.66 ± 7.70		91.29 ± 10.50	

**Table 3:** Univariate and multivariate (backward stepwise conditional method) Cox regression analysis for factors associated with overall survival (OS).

	Univariate Analysis		Multivariate Analysis	
	cHR (95% CI)	*p* Value	aHR (95% CI)	*p* Value
Age				
<65	REF.			
≥65	2.67 (1.59–4.47)	<0.001	–	
Performance status				
90–100%	REF.		REF.	
<90%	4.22 (2.50–7.13)	<0.001	2.96 (1.70–5.17)	<0.001
ASA class				
I	REF.			
II–III	3.07 (1.83–5.14)	<0.001	–	
Tumor volume				
Small	REF.			
Large	2.99 (1.20–7.47)	0.019	–	
CC-score				
CC-0	REF.			
CC-1	1.93 (0.88–4.26)	0.103	–	
Histopathology				
Serous	REF.		REF.	
Other	0.10 (0.03–0.42)	0.002	0.14 (0.03–0.59)	0.007
Degree of differentiation				
G1/G2	REF.			
G3	6.89 (2.49–19.03)	<0.001	–	
Lymph node dissection				
Conventional	REF.			
RLNR	0.72 (0.35–1.46)	0.357	–	
PCI				
0–13	REF.			
14–20	2.33 (1.26–4.31)	0.007	–	
21–39	4.50 (2.40–8.44)	<0.001	–	
Morbidity				
No	REF.		REF.	
Yes	3.53 (2.10–5.93)	<0.001	6.87 (3.49–13.52)	<0.001
HIPEC				
No	REF.		REF.	
Yes	0.60 (0.35–1.00)	0.051	0.33 (0.17–0.63)	0.001

cHR = crude hazard ratio; aHR = adjusted hazard ratio; CI = confidence interval.

**Table 4:** Univariate and multivariate (backward stepwise conditional method) Cox regression analysis for factors associated with disease-specific survival (DSS).

	Univariate Analysis		Multivariate Analysis	
	cHR (95% CI)	*p* Value	aHR (95% CI)	*p* Value
Age				
<65	REF.			
≥65	2.18 (1.01–4.74)	0.048	–	
Performance status				
90–100%	REF.			
<90%	5.29 (2.37–11.82)	<0.001	–	
ASA class				
I	REF.			
II–III	3.64 (1.66–7.99)	0.001	–	
Tumor volume				
Small	REF.			
Large	2.07 (0.62–6.91)	0.236	–	
CC-score				
CC-0	REF.			
CC-1	2.23 (0.67–7.47)	0.194	–	
Histopathology				
Serous	REF.			
Other	0.10 (0.02–0.74)	0.024	–	
Degree of differentiation				
G1/G2	REF.		REF.	
G3	15.05 (2.04–111.19)	0.008	8.64 (2.03–36.73)	0.003
Lymph node dissection				
Conventional	REF.			
RLNR	0.37 (0.09–1.56)	0.174	–	
PCI				
0-13	REF.			
14-20	1.59 (0.64–3.95)	0.315	–	
21-39	3.14 (1.12–8.81)	0.030	–	
Morbidity				
No	REF.			
Yes	0.79 (0.24–2.63)	0.695	–	
HIPEC				
No	REF.		REF.	
Yes	0.25 (0.09–0.65)	0.005	0.25 (0.10–0.63)	0.003

cHR = crude hazard ratio; aHR = adjusted hazard ratio; CI = confidence interval.

**Table 5:** Univariate and multivariate (backward stepwise conditional method) Cox regression analysis for factors associated with disease-free survival (DFS).

	Univariate Analysis		Multivariate Analysis	
	cHR (95% CI)	*p* Value	aHR (95% CI)	*p* Value
Age				
<65	REF.			
≥65	1.44 (0.80–2.57)	0.222	–	
Performance status				
90–100%	REF.			
<90%	2.52 (1.32–4.81)	0.005	–	
ASA class				
I	REF.			
II–III	1.94 (1.03–3.63)	0.040	–	
Tumor volume				
Small	REF.			
Large	2.28 (0.97–5.37)	0.059	–	
CC-score				
CC-0	REF.			
CC-1	1.05 (0.33–3.40)	0.929	–	
Histopathology				
Serous	REF.			
Other	0.15 (0.05–0.49)	0.002	–	
Degree of differentiation				
G1/G2	REF.		REF.	
G3	4.58 (1.94–10.79)	0.001	4.13 (1.69–10.13)	0.002
Lymph node dissection				
Conventional	REF.			
RLNR	0.74 (0.35–1.58)	0.434	–	
PCI				
0–13	REF.		REF.	
14–20	1.50 (0.75–2.97)	0.250	1.17 (0.57–2.41)	0.135
21–39	3.38 (1.65–6.91)	0.001	2.32 (1.10–4.89)	<0.001
Morbidity				
No	REF.			
Yes	1.55 (0.77–3.11)	0.221	–	
HIPEC				
No	REF.		REF.	
Yes	0.66 (0.38–1.17)	0.152	0.54 (0.30–0.97)	0.041

cHR = crude hazard ratio; aHR = adjusted hazard ratio; CI = confidence interval.

## Data Availability

The datasets presented in this article are not readily available as they are part of an ongoing study.
